# Delay in healthcare seeking and associated factors among patients presenting with sexually transmitted infection symptoms in the Horo Guduru Wollega Zone, Oromia, Western Ethiopia, 2022

**DOI:** 10.3389/frph.2024.1348262

**Published:** 2024-07-08

**Authors:** Getachew Abebe Guddu, Ayantu Getahun, Girma Yadesa, Tadesse Tolossa

**Affiliations:** ^1^ Disease Prevention and Control Core Process Coordinator, Horro Guduru Wollega Zonal Health Office, Shambu, Ethiopia; ^2^ Department of Public Health, Institutes of Health Sciences, Wallaga University, Nekemte, Ethiopia; ^3^ Nursing Department, College of Medicine and Health Sciences, Diredawa University, Diredawa, Ethiopia

**Keywords:** delay, healthcare seeking, sexually transmitted infections, Horo Guduru Wollega Zone, STIs

## Abstract

**Background:**

Sexually transmitted infections (STIs) are the most prevalent communicable diseases that impact people's health and wellbeing. One of the main obstacles to successful prevention and control of STIs is the delay in seeking healthcare, which can result in significant personal and economic losses. However, there are limited studies on the delay in healthcare seeking among STI patients in resource-limited countries such as Ethiopia.

**Objective:**

This study aims to assess delays in seeking healthcare and associated factors among patients presenting with STIs at public health facilities in the Horo Guduru Wollega Zone, Oromia, Western Ethiopia, 2022.

**Methods:**

A cross-sectional study was conducted at public health facilities in the Horo Guduru Wollega Zone from 15 March to 15 May 2022. The study included 507 participants and used a consecutive sampling method. The outcome variable of the study was the delay in seeking healthcare among STI patients. A binary logistic regression model was used to identify candidate variables with a *p*-value of less than 0.25 after analyzing each variable separately. Finally, a multivariable analysis was performed to determine statistically significant variables at a *p*-value of less than 0.05, and an adjusted odds ratio (AOR) was reported.

**Results:**

The proportion of individuals with STIs who delayed seeking healthcare in the Horo Guduru Wollega Zone was 61.3% [95% confidence interval (CI): 57%–65.5%]. Factors such as age >35 years (AOR = 2.23, 95% CI: 1.26–3.95), higher educational level (AOR = 2.72, 95% CI: 1.55–4.74), lack of condom use (AOR = 1.63, 95% CI: 1.05–2.55), and travel time to health facilities of >1 h (AOR = 4.30, 95% CI: 1.70–10.89) were found to be significantly associated with delayed healthcare seeking.

**Conclusion and recommendations:**

This study found that the magnitude of delay in seeking healthcare was higher than the national average and identified several contributing factors. Interventions such as developing educational programs and improving access to healthcare services are crucial for supporting patients with STIs. Enhancing healthcare accessibility in rural areas and promoting the use of condoms through targeted community outreach can reduce travel time and prevent delays in seeking healthcare for STIs.

## Introduction

One of the major challenges in preventing and controlling sexually transmitted infections (STIs) is the delay in seeking healthcare, which can lead to complications such as pelvic inflammatory disease, ectopic pregnancy, infertility, cervical cancer, fetal loss, adverse birth outcomes, and increased risk of human immunodeficiency virus (HIV) infection (1). Delaying the treatment of infection can prolong its duration and increase the likelihood of STI transmission by up to 10 times, resulting in mortality and infertility (2–4). This can also increase the incidence of STIs by broadening the time between the onset of infection and treatment, hindering the successful prevention and control of the infections, and resulting in significant personal and economic loss (3, 5).

The percentage of individuals who postpone seeking treatment for STIs varies widely, ranging from 23% to 73% in both developed and developing countries (6). According to studies conducted in various regions of Africa, a significant percentage of patients presenting with STIs experience delays in seeking healthcare. For instance, the figure stands at 42% in Laos (7), 64% in Durban (6), and58% in Uganda (8).

The World Health Organization (WHO) recommends a holistic approach to STI management: treat, cure, reduce infectiousness, minimize complications, and prevent risky behavior. Strategies include pre-exposure prophylaxis (PrEP), voluntary medical male circumcision, and ensuring partners receive proper treatment (9). Ethiopia's 2015–2020 HIV/AIDS Strategic Plan focuses on improving program management, establishing structures, and allocating staff. It targets healthy sexual behavior, condom use promotion, and treatment-seeking behavior through behavior change communication. The plan enhances the capacity of healthcare providers for STI diagnosis, treatment, counseling, and reporting. It expands STI services to vulnerable populations and prevents congenital syphilis through routine rapid plasma regain (RPR) testing in all antenatal care (ANC) clinics (10, 11).

Ethiopia is working to improve the quality and accessibility of healthcare services by increasing the number of health facilities (HFs) to achieve 100% geographical coverage and incorporating all community-based health insurance (CBHI) beneficiaries into its health sector development plan while also adhering to the WHO guidelines (10, 12). Due to delays in seeking healthcare, opportunities for managing STIs, such as the implementation of comprehensive interventions, including treatment, counseling, and the promotion of condom use, were missed for affected individuals. Although not specific to individual cases, the 2021 Zonal Health Office report for Horo Guduru Wollega (HGW) reveals an annual per-capita outpatient department (OPD) rate of 0.6, which is below the national standard (13).

Research suggests that socio-demographic, individual, and institutional factors can contribute to delays in seeking healthcare for STIs. Specifically, studies indicate that 66% of patients with STIs experience delays in seeking healthcare due to individual factors such as having two or more sexual partners and fear of stigma as barriers to treatment. In addition, 49.4% of patients with STIs report institutional factors, such as lack of access to healthcare services, as a contributing factor to delays in seeking healthcare (14, 15). Despite its public health importance, the evidence regarding delays in seeking healthcare among patients with STIs is limited. Hence, the study aimed to assess delays in seeking healthcare and associated factors among patients presenting with STI in the Horo Guduru Wollega Zone, Oromia, Western Ethiopia.

## Methods

### Study area and study design

A cross-sectional study was carried out in the public health facilities of the Horo Guduru Wollega Zone from 15 March to 15 May 2022. The Horo Guduru Wollega Zone is one of the 21 zones in the Oromia National Regional State in western Ethiopia, with 13 districts. The zone is located 314 km away from Addis Ababa, the capital city of Ethiopia. The study was conducted in 13 health centers and three government hospitals that serve as antiretroviral therapy (ART) sites for STIs, and these facilities contributed 56% of the cases reported in the zone over a 3-month period.

### Population

The study included all STI patients seeking care in selected public health facilities within the Horo Guduru Wollega Zone from 15 March to 15 May 2022. Symptomatic cases diagnosed by syndromic (*n* = 461) and laboratory (*n* = 42) methods were considered (*n* = 507). The source population encompassed all STI patients in the zone, while the study population consisted of those seeking care during the specified period. Critically ill and psychiatric patients were excluded.

### Data collection tools and procedures

The questionnaire used to collect the data was adapted from various literature sources on delays in seeking healthcare (1, 16, 17) and existing guidelines and was subsequently modified to fit the context of the study. It included questions on socio-demographic, individual, and institutional factors. The data were collected from eligible patients with STIs who were presented to public health facilities between 15 March and 15 May 2022. The outcome variable was the delay in seeking healthcare among these patients (recorded as “Yes” if the patient presented to the health facility 7 or more days after the onset of symptoms and “No” if the patient presented within 7 days of symptom onset). Independent variables included socio-demographic factors, such as age, sex, marital status, educational status, occupational status, family income, residence, ethnicity, and religion of the patients; individual factors, such as the source of information, duration of symptoms, condom use, sexual debut, and number of sexual partners; and institutional factors, such as distance of the health facility from the patient’s home, travel time, and means of transportation. Two trained health officers and a BSc Nurse working in public health facilities for each randomly selected health institution were recruited for data collection.

### Data quality assurance

Afan Oromo translations of the questionnaires were conducted with the assistance of a language expert, and these were later translated back to English. The principal investigator provided a 1-day training session for the data collectors, with close supervision maintained throughout the data collection process. Prior to actual data collection, a pre-test was conducted on a sample of 5% of patients with STIs to evaluate the clarity of the questionnaire. Based on the pre-test results, necessary amendments to the questionnaire were made.

### Data management and analysis

The data entry for this study was performed using Epi data version 3.2, and the data were subsequently exported to SPSS version 25 for further analysis. Prior to analysis, the data were cleaned and edited by utilizing simple frequencies and cross-tabulation. The categorical variables were recategorized, and continuous variables were categorized to make them suitable for analysis. Descriptive statistics, including frequencies, percentages, mean, and standard deviation, were calculated. The chi-squared test was used to check assumptions for logistic regression. A binary logistic regression model was employed to identify factors associated with delays in seeking healthcare among patients with STIs. Factors significant at a 25% level (*P*-value ≤ 0.25) in the bi-variable logistic regression analysis were included in the multivariable logistic regression analysis using a forward stepwise selection process. The final multivariable logistic regression table presented the crude (COR) and adjusted odds ratios (AOR) and their corresponding 95% confidence intervals (CIs). AOR with 95% confidence intervals was computed, and statistical significance was declared when it was significant at a 5% level (*p*-value < 0.05).

### Ethical approval

Ethical approval was obtained from Wallaga University, Institute of Health Sciences, with support from the Horo Guduru Wollega Zonal Health Office. Permission letters were issued to selected health facilities. Patients received an explanation of the purpose of the study before providing informed written consent. Interviews were conducted in private rooms to ensure confidentiality. The questionnaire excluded personal identifiers. Participants were assured that choosing not to participate would not result in any penalties and their responses would have no impact on their care.

## Results

### Socio-demographic characteristics of STI patients

Between 15 March and 15 May 2022, 507 STI patients in Horo Guduru Wollega public health facilities were interviewed, aged ranging from 15 to 48 years (mean: 29.02, SD: ± 6.74). Most participants (43.8%) were aged 25–34 years, 59.2% were married, and 30% were merchants. The study included a predominantly Oromo population (91.7%), with 53.3% identifying as Protestant followers, and nearly 66% residing in urban areas (Table 1).

**Table 1:** Socio-demographic characteristics of patients with STIs presenting to public health facilities in the Horo Guduru Wollega Zone, Oromia, Western Ethiopia, 2022.

Variables	Response	Frequency (*n* = 507)	Percent
Sex	Male	245	48.3
	Female	262	51.7
Age (years)	15–24	151	29.8
	25–34	222	43.8
	>35	134	26.4
Ethnicity	Oromo	465	91.7
	Amhara	27	5.3
	Others	15	3.0
Marital status	Single	173	34.1
	Married	300	59.2
	Divorced	29	5.7
	Widowed	5	1.0
Educational status	No formal education	92	18.1
	Primary	91	17.9
	Secondary and higher	324	63.9
Residence	Urban	333	65.7
	Rural	174	34.3
Occupational status	Farmer	96	18.9
	Government employed	130	25.6
	Merchant	152	30.0
	Student	129	25.4
Religion	Protestant	270	53.3
	Orthodox	168	33.1
	Muslim	39	7.7
	Wakefata	30	5.9

### Individual-related factors

Among study participants, 378 (74.6%) did not use condoms with their sexual partners, despite 235 (46.4%) reporting having more than two sexual partners in the last 12 months. In addition, more than 30% of the participants started having sexual intercourse before the age of 16 years (Table 2).

**Table 2:** Individual-related factors among patients with STIs presenting to public health facilities in the Horo Guduru Wollega Zone, Oromia, Western Ethiopia, 2022.

Variable	Response	Frequency (*n* = 507)	Percent
Sexual debut (years)	<16	176	34.7
	≥16	331	65.3
Condom use	Yes	129	25.4
	No	378	74.6
Number of sexual partners	One	272	53.6
	Two and above	235	46.4
Duration of symptoms (days)	<7	196	38.7
	≥7	311	61.3

### Institutional-related factors

Among study participants, the majority, 450 (88.8%), can access healthcare services within 10 km, with only 45 (9%) traveling more than 1 h to reach a health facility. Nearly 60% of the participants are members of community-based health insurance (Table 3).

**Table 3:** Institutional-related factors among patients with STIs presenting to public health facilities in the Horo Guduru Wollega Zone, Oromia, Western Ethiopia, 2022.

Variable	Response	Frequency (*n* = 507) (%)	Percent
Distance from HF (km)	<10	450	88.8
	≥10	57	11.2
Means of transport to HF	On horseback	26	5.1
	On foot	268	52.9
	By minibus	57	11.2
	By Bajaj	159	31.4
Approach of HCPs	Caring HCPs	278 (54.8)	159
	Unfriendly HCPs	186 (36.7)	120
	Neutral	43 (8.5)	32
Travel time (min)	<60	462	91.1
	≥60	45	8.9
CBHI	Member	300	59.2
	Not member	207	40.8

CBHI, community-based health insurance; HCP, healthcare professional.

### Magnitudes of delays in seeking healthcare

**Figure 1:**
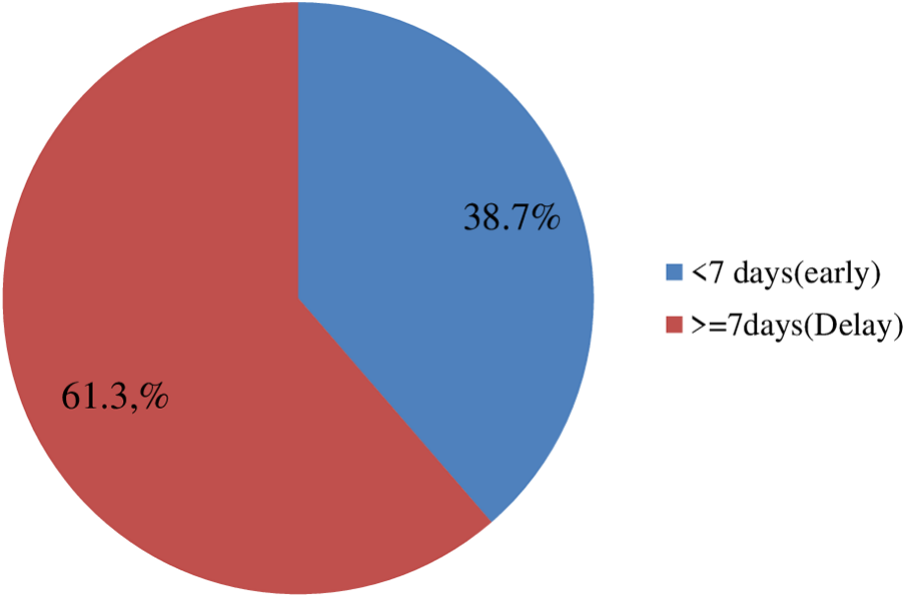
Magnitude of delay in seeking healthcare among patients presenting with STIs to public health facilities in the Horo Guduru Wollega Zone, Oromia, Western Ethiopia, 2022.

**Figure 2:**
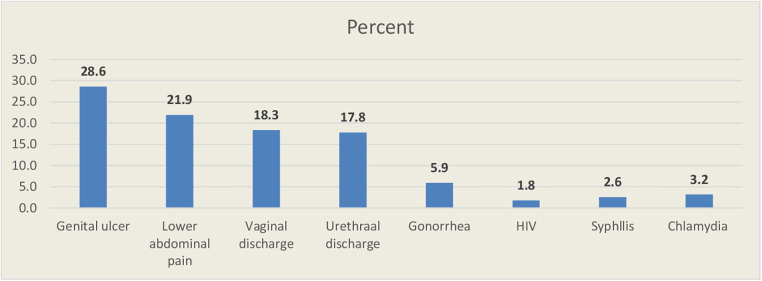
Types of STIs diagnosed by laboratory and syndromic methods among patients with STIs presenting to public health facilities in the Horo Guduru Wollega Zone, Oromia, Western Ethiopia, 2022.

In this study, 311 participants (61.3%, 95% CI: 57–65.0) delayed seeking healthcare, presenting to health facilities 7 days after noticing the signs and symptoms of an STI, with a mean delay time of 14.9 ± 18.461 (Figure 1). About 46 (9.1%) patients were diagnosed through laboratory testing and 461 (90.9%) were diagnosed based on syndromes. Genital ulcers (28.6%), lower abdominal pain (21.9), and vaginal discharge (18.3%) were the most reported STI symptoms (Figure 2).

### Factors associated with delays in seeking healthcare

After conducting bi-variable analysis, several variables were identified as potential candidates for multivariable logistic regression analysis, and their significance in the model was assessed. After adjusting for other variables in the model, the likelihood of delays in seeking healthcare was nearly two times higher among patients with STIs aged >35 years than those aged 15–24 years (AOR = 2.23, 95% CI: 1.26–3.95). Those with secondary or higher education were 2.72 times more likely to delay seeking healthcare than those without formal education (AOR = 2.72, 95% CI: 1.55–4.74). Patients who traveled for more than 1 h for healthcare were 4.30 times more likely to delay seeking healthcare than those who traveled for 1 h or up to 1 h (AOR = 4.30, 95% CI: 1.70–10.89). The odds of delaying healthcare were 1.63 times higher among patients who never used condoms with their sexual partners than those who did use condoms (AOR = 1.63, 95% CI: 1.05–2.55) (Table 4).

**Table 4:** Multivariable logistic regression analysis to identify factors associated with delayed healthcare seeking of patients presenting with STIs to public health facilities in the Horo Guduru Wollega Zone, Oromia, Western Ethiopia, 2022.

Variable	Response	Healthcare seeking		COR (95% CI)	AOR (95% CI)	*p*-value
		Delay *n* (%)	Early *n* (%)			
Age (years)	≥35	95 (71)	39 (29)	1.74 (1.07–2.86)	2.23 (1.26–3.95)	0.006*
	25–34	128 (58)	94 (42)	0.97 (0.64–1.48)	1.02 (0.64–1.62)	0.943
	15–24	88 (58)	63 (42)	1		
Educational status	Secondary and higher	213 (66)	111 (34)	1.54 (0.96–2.40)	2.72 (1.55–4.74)	0.001*
	Primary	47 (52)	44 (48)	0.86 (0.48–1.54)	0.99 (0.52–1.89)	0.992
	No formal education	51 (55)	41 (45)	1		
Condom use	No	245 (65)	133 (35)	1.76 (1.17–2.64)	1.63 (1.05–2.55)	0.030*
	Yes	66 (51)	63 (49)	1		
Travel time (min)	≥60	39 (87)	6 (13)	4.54 (1.89–10.94	4.30 (1.70–10.89	0.002*
	<60	272 (59)	190 (41)	1		
Approaches of HCPs	Neutral	32 (74)	11 (26)	2.18 (1.05–4.49)	2.04 (0.97–4.29)	0.062
	Unfriendly	120 (64)	66 (36)	1.36 (0.93–1.99)	1.14 (0.76–1.72)	0.532
	Caring	159 (57)	119 (49)	1		
CBHI	Member	187 (62)	113 (38)	1.12 (0.77–1.59)	1.18 (0.75–1.86)	0.505
	Not member	124 (60)	83 (40)	1		
Distance (km)	≥10	39 (68)	18 (32)	1.42 (0.79–2.56)	1.23 (0.61–2.48)	0.446
	<10	272(60)	178(40)	1		

HCP, healthcare professional.

* Statistically significant at *p* < 0.05.

## Discussion

The purpose of this study was to assess the delay in seeking healthcare and associated factors among patients presenting with STIs in public health facilities of the HGW Zone. The study found that 311 (61.3%) patients presented to health facilities after 7 days of the onset of symptoms. This proportion of delayed healthcare seeking was higher than the national level report from the 2016 Ethiopia Demographic and Health Survey (EDHS) (2) and studies conducted in the towns of Gambella (1) and Adama (16). This difference may be due to variations in sample size and area of study or the difference in study population. In addition, the method used to diagnose STIs might account for the differences observed. Most previous studies utilized laboratory testing to investigate STIs, whereas our study primarily employed the syndromic approach. However, this study reported a lower magnitude of delay in seeking healthcare than a study conducted in Durban, South Africa (6). This difference might be due to variations in the study area or the difference in awareness regarding the importance of early healthcare seeking in South Africa and Ethiopia.

This study indicates that individuals aged ≥35 years were twice as likely to delay seeking healthcare compared to those aged 15–24 years. This finding is consistent with a previous study conducted in Kenya (18). This might be due to the fact that older individuals often have more family and work responsibilities, making it harder to prioritize their own health compared to younger populations. In addition, the older population might perceive the symptoms as minor problems, and fear of stigma might also contribute to delays in seeking healthcare (19). Nevertheless, this result contradicts a Nepalese study that found being over 35 years old to be protective against delays in seeking healthcare (20). The discrepancy could be due to differences in the study areas or cultural and normative variations among the participants.

Those with secondary and higher education delay in healthcare seeking twice as much as those who with no formal education. This could be because individuals with formal education may have developed confidence in managing illnesses at home, which could lead to delayed healthcare seeking. In addition, uneducated individuals might have acquired health literacy outside of formal education, contributing to more proactive healthcare seeking.

This study revealed that the number of patients with STIs who never use a condom and delay healthcare seeking is 1.63 times higher than those who use a condom with their sexual partners. This is consistent with the study conducted in Durban, South Africa (6). This could be due to a lack of awareness, as those who do not use condoms may also lack awareness regarding the importance of early treatment for STIs, resulting in delayed healthcare seeking. Again, this study depicted that patients with STIs who presented to HFs after about 1 h of travel time were four times more likely to delay healthcare seeking than those who traveled for less than 1 h to reach HFs; this is in line with a study done in Kenya (8). The reason for delayed healthcare-seeking behavior of this population could be attributed to lack of transportation, inaccessibility of health facilities, time constraints, and financial constraints preventing travel over long distances.

### Limitation and strength of the study

The study attempted to generalize the healthcare seeking behavior by using large sample sizes from many sites. There might be a recall bias, as most of the information collected from patients pertains to their sexual experiences within the previous year. The study used a cross-sectional study design, which cannot show cause–effect relationships.

## Conclusion

This study reported a higher magnitude of delay in seeking healthcare than the national magnitude, suggesting that there is a chance for an increased duration of infectiousness in the Horo Guduru Wollega Zone. Factors such as age ≥35 years, secondary and higher education level, sexual intercourse without a condom, and travel time to reach health facilities of >1 h were statistically associated with delay in seeking healthcare among patients with STIs in the HGW Zone. Interventions such as developing educational programs and improving access to healthcare services are crucial for supporting patients with STIs. Enhancing healthcare accessibility in rural areas and promoting condom use through targeted community outreach can reduce travel time and prevent delays in seeking healthcare for patients with STIs.

## Data Availability

The raw data supporting the conclusions of this article will be made available by the authors without undue reservation.
